# The Annual Meeting of the B.M.A. at Brighton

**Published:** 1913-08-02

**Authors:** 


### THE NATIONAL MEDICAL GUILD.

### The Annual Meeting of the B.M.A. at Brighton.

As we promised in the last article, we devote this week to an examination of the results of the meeting of the representative body of the British Medical Association. That these decisions will have a far-reaching effect on the future of the Association no one who has studied the official report of the meeting can deny. In the first place, the Association has definitely rejected the principles of trade unionism, at any rate for one more year, the motion to postpone the consideration of this project until next representative meeting being passed. The arguments in favour of unions being ably presented to the meeting by Dr. Wallace Henry, of Leicester, the chairman of the Leicester Union of Practitioners, who dwelt on the one point that the British Medical Association seems incapable of understanding, viz., the security accorded to the funds of any trade union and the immunity from attachment of those funds. The importance of this point the solicitors to the Association and the State Sickness Insurance Committee are fully aware of, as they mention in their report on the "Trust Fund" scheme that any fund raised in the manner that they propose would be liable to attachment at the exact moment that it was required for the defence of the profession. And with that statement ringing in their ears the representatives deliberately proceed to vote 107 against and 53 for the amendment. The ostrich with his head in the sand is an intelligent animal in comparison! However, it is satisfactory that there are fifty-three members of the representative body who have embraced the views that the Guild hold, and on the principle that a little leaven leaveneth the whole lump, the Association as a body may speedily adopt the only safe course open to them.

The main arguments against the amendment were sentimental. Dr. Macdonald reminded the meeting that medical men were gentlemen by Act of Parliament, and he was sure that no gentleman could ever become a member of a trade union. Putting aside the fatuity of the argument, Dr. Macdonald has the misfortune to differ from the leaders of the profession in other countries where the experiment has been tried. Germany disliked unionism until the machinations of the Socialist party forced them to adopt the principle and to shield themselves behind those privileges that only a trade union can afford.

Let the mind of the profession be clear once and for all on the subject, and at the risk of being reiterative the writer repeats the arguments that were made use of in a former issue of this Journal. In the first place, do the profession think that the safety from attacks in the future rests on the establishment of a large fighting fund which can be used to help them in the hour of need? Will Dr. Macdonald and the opponents of trade unionism say whether they want a defence fund or not? Personally, we think that they do, and as evidence of the truth of that surmise we have the report of the State Sickness Insurance Committee, which recommends that a subscription of £5 per annum per person be paid into a central defence fund, there to accrue until the profession needed it. Surely this is sufficient evidence that there is a need of a fund.

In the second place, does not the Council say that the absence of such a fund was largely responsible for the debacle of December and January last, and are they not right?

Finally, will not everyone, trade unionist or not, agree that if such a fund be raised it must be kept intact and inviolate, and, what is of even greater importance, that it be kept SAFE?

To sum up, therefore, we will leave to the profession as the jury in this matter three questions, viz.:—

1. Was the debacle of last year the direct result of the want of money?

2. Do the British Medical Association desire to raise a fund in order to be ready for the next fight?

3. Do the British Medical Association and every medical man, whether a trade unionist or not, desire that the fund so raised shall be protected from any chance of its being attached when it is most wanted?

If the answer of the profession to these three questions be in the affirmative, as we think that it must be, we can proceed to advance the argument a step further.

On page 29 of the Supplement to the *British Medical Journal* of July 5, 1913, occur the following words:—

"The risks run by the Trust would be that in carrying out the objects of the Fund . . . it would be open to the charge that it was engaged in activities that might be held to be in restraint of trade. TRUST FUNDS CANNOT CLAIM ANY LEGAL PROTECTION IN DOING SUCH THINGS. THE ONLY ORGANISATION WHICH HAS EFFECTIVE LEGAL PROTECTION IN SUCH ACTIONS IS A TRADE UNION."

In the face of this opinion in the Journal of the Association, and the vote taken at the representative meeting, we may be excused in wondering, in company with hundreds of other medical men, where the consistency of the Association comes in. Deliberately to throw away for another precious twelve months the only "effective legal protection" (the words are their own) that they have is a piece of folly that would be laughable if it were not so important, and the writer ventures to think that the folly shown by the representatives who voted against the amendment is criminal.

The net result of all this is that the National Medical Guild, instead of working hand-in-hand with the Association as its promoters wished, will inevitably be driven to leave the Association to that "gentlemanly" policy of *laisser faire* that it has followed with such conspicuous success in the past, and that it seems determined to follow in the future.

The Association, by its rejection of the trade union amendment, has marked out the path along which the Guild will travel. We shall go on and go forward, securing adherents where we can, and steadily raising and setting on one side a defence fund for the use of our members; a fund which shall be free from the enormous administration charges proposed by the State Sickness Insurance Committee, and free also from the attacks of the enemy when next it becomes necessary to fight. Further, we appeal to all those gentlemen and their constituents who voted for the amendment to come over and help us, in order that we may have that strength that numbers alone can give.

